# Systolic and Diastolic Dysfunction of the Right Heart Ventricle in Patients With Chronic Obstructive Pulmonary Disease in Extremely Cold Climate

**DOI:** 10.5539/gjhs.v7n3p283

**Published:** 2014-11-30

**Authors:** Anna Innokentievna Sivtseva, Andrew Vladislavovich Strutynsky, Vadim Grigorievich Krivoshapkin, Elena Nikolaevna Sivtseva, Marianna Adolfovna Ivanova, Leonid Fedorovich Timofeev

**Affiliations:** 1 NI Pirogov Russian National Research Medical University, Ostrovitianov str. 1, Moscow 117997, Russia; 2 MK Ammosov North - Eastern Federal University, Belinskiy str. 58, Yakutsk 677000, Russia

**Keywords:** echocardiography, chronic obstructive pulmonary disease, chronic cor pulmonale, remodeling

## Abstract

The paper describes echocardiographic values of systolic and diastolic dysfunction the right heart ventricle in 229 patients with chronic obstructive pulmonary disease. In our patients the values AvPAP (≥25 mmHg while resting), FDDrv and FSDrv (>26 and 20 mm respectively), the thickness of front wall the RV (>5 mm), the dimension of AD (>35 mm), as well as the reduction the vestibular-distal shortening of RV (<23%), maximum blood velocity and the blood evacuation time from reflect indirectly the progressive reduction the contractive capacity RV myocardium and the occurrence of systolic dysfunction. In patients with severe de-compensation a restrictive type diastolic function is more characteristic – acceleration of early diastolic filling and blood velocity decrease during the auricular systole.

## 1. Introduction

Currently there is a commonly known hypothesis explaining the progressive character of the remodeling and formation of the chronic heart failure (CHF) being the neuro-hormonal heart failure model (Bokeria, Plakhova, Ivanitsky, & Gorbachevsky, 2001; Brunova & Ergeshov, 2002; Fyodorova, Khimochko, & Roytman, 2001; Chernyayev, Lebedin, Dulin, Mikhalyova, Samsonova, Vertikova, & Bazarov, 2005; Global Initiative for Chronic Obstructive Lung Disease, 2013; Schiavon et al., 2012; Richens & Howard, 1983; Wang, Yang, Guo, Uyeminami, Dong, Hammock, & Pinkerton, 2011; Quon, Gan, & Sin, 2008; Wrobel, Thompson, & Williams, 2011; Pfeffer et al., 1992; Xing, Xy, Shi, & Du, 2012; Richard & Devereux, 1995; Belenkov, 2002), and stating the remodeling of the heart being the actuation of several neuro-endocrine systems, whereof the most important are: SAS, RAAS (kidneys–adrenal glands), tissue RAS, auricular Na-urethic peptide, endothelial dysfunction (endotheline, reduction of nitrogen oxide synthesis, prostacycline), tumor necrosis factors etc. The effects thereof result in actuation of endocellular fermenting systems (cininases, phosphatases, etc.) and a launch of transcription factors immediately influencing the expression of the promoters (genes) and activating heart re-modeling processes (Vikentyev, 2001; Zubritsky, 2002; Mazur, 2001; Bernardzhevskaya, 2005; Strutynsky, Glazunov, & Reissner, 2000)

**Figure 1:**
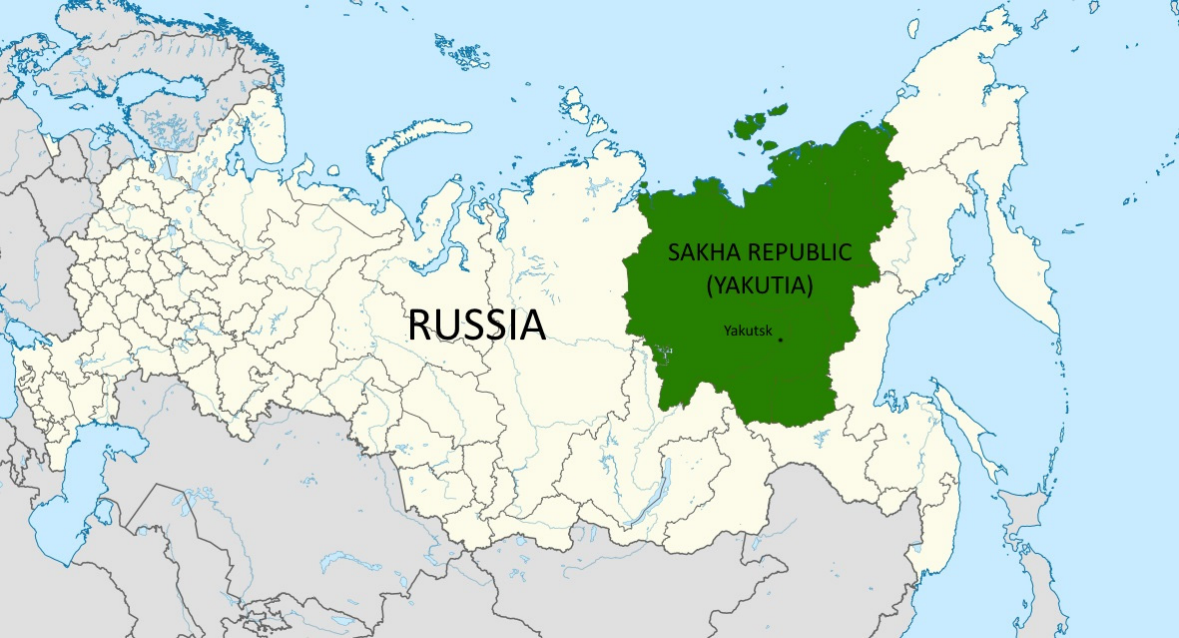
Map of the Republic Sakha (Yakutia)

The purpose of the present paper was to detect the formation of chronic cor pulmonale in patients with COPD, living in the Central Area of Sakha Republic (Yakutia) (Figure 1).

## 2. Materials and Methods

The basis of this part of the paper was formed by the results of clinical lab and instrumental examination of 229 COPD patients, in cases of complicated CCP.

COPD was determined after generally accepted criteria (Schiavon et al., 2012; Richens & Howard, 1983; Tailor, 2006; Menshikova, 1996) based on the typical clinical finding of the disease (prolonged cough with sputum, intermitting fever, gradually growing anhelation with decreasing tolerance to muscular strain, retained beyond acute periods of the disease, a characteristic auscultatory finding in the lungs), anamnesis data (typical disease exacerbations), x-ray findings (obstructive pulmonary emphysema, pneumosclerosis, changes of the lungs pattern), as well as the results of the external breath examination (obstructive or mixed type of RI with progressive FEP_1_). This, for all examined patients a combination of bronchial obstructive syndrome and lesions of respiratory pulmonary segments. Partially (124 cases), signs of decompensated CCP and PAH (after clinical and echocardiographic data) were evident.

Thus, the main inclusion criteria of the patients in this part of the investigation were:

1) Available trustworthy clinical and instrumental signs of COPD (chronic cough, sputum secretion, progressive anhelation);
2) FEP1/FVCL less than 70% of the norm;
3) Patient’s informed consent.

The severity of the disease was determined after GOLD classification, rev. 2013

The diagnostic criteria of chronic cor pulmonale were:

1) Generally accepted clinical and instrumental signs of RV hypertrophy and/or widening of the hollow of RV and AD;
2) Echocardiographic signs of AvPAP increase >20 mmHg;
3) Clinical signs of blood congestion in the greater circulation (megalohepatia, oedemata of lower extremities, swelling of neck veins, hydrothorax, ascites, refluxus hepatojugularis etc.);
4) Echocardiographic signs of systolic dysfunction of RV.

It should be underlined, that in each particular case the clinical and instrumental diagnosis of CCP was based on meeting of 2 or more criteria, as described above.

The exemption criteria of patients from the group were:

- Distinct signs of bronchospasm and frequent asphyxia attacks in patients with accompanying bronchial asthma.
- Detection of acute MI or unstable angina pectoris.

The clinical and instrumental examination of COPD and CCP patients was conducted during the period of COPD relevant emission and in absence of the intensification of the inflammation process in the lungs and bronchi.

All patients were divided into 3 groups. The 1^st^ group was formed by 105 COPD patients without trustworthy signs of CCP. The 2^nd^ group comprised 71 COPD patients with compensated CCP signs. The 3^rd^ group included 53 patients with de-compensated CCP and CHF of RV signs.

The data obtained were statistically processed.

## 3. Echocardiography and Doppler Echocardiography

The echocardiography was performed with the echocardiographic unit “Acuson-128 XP” (USA). The instrument ensures ultrasonic investigation in 2D and M-Mode, as well as the Doppler investigation of the blood flux in pulse and constant wave modes. The investigation was arranged after a generally accepted method using the left parasternal, apical and subcostal accesses.

The investigation was conducted simultaneously with ECG recording to synchronize the phases of the heart cycle of the echocardiogram with the ECG data. The wall thickness and heart hollows dimension measurement in systolic and diastolic phases were conducted in accordance with the method of the American Experts Committee on Echocardiography. The residual volumes of the left ventricle (FDV and FSV), the stroke volume (SV) and the discharge fraction (DF) were calculated after Simpson. The average pressure of the PA (AvPAP) was determined after A. Kitabatake (1983), whereas the quantitative parameters of the systolic blood flow in the outlet segment of the RV were measured and the flow acceleration time (AcT) to the total duration of the expulsion from RV (RVET) was calculated.

The diastolic function of the RV and LV were evaluated after the results of the Doppler measurement, accordingly, of those of trans tricuspid and transmission diastolic blood flow in pulse, color and constant wave modes.

## 4. Investigation Results and Discussion

Table 1 presents the results of a comparative analysis of abnormalities of systolic and diastolic function of the RV, as well as the alteration of the average pulmonary artery pressure (AvPAP) in COPD and CCP patients. It is obvious that the patients of the 1^st^ group (without generally accepted CCP signs) only a statistically non-trustworthy tendency (p>0.05) to a slight increase of the AvPAP is observed as compared with the reference group patients. A statistically significant increase of this hemodynamic parameter was detected only in the patients of the 2^nd^ and the 3^rd^ group with verified CCP (p <0.001). Thereby the AvPAP level in the patients of the 3^rd^ group with de-compensated CCP proved in general to be higher than in CCP patients of the 2^nd^ group without CHF signs (p <0.05). Nevertheless, the pressure increase of the PA in the patients of the 2^nd^ and the 3^rd^ group was moderate, and in never exceeded 22.6±1.2 and 27, 4±1.2 mmHg, respectively.

**Table 1:** Echocardiographic signs of systolic and diastolic dysfunction of the right heart ventricle in COPD and CCP patients

	COPD patients			COPD patients		

Parameters	1st group (no CCP)	2nd group (CCP comp.)	P1-2	2nd group (CCP comp.)	3rd group (CCP de-comp.)	P2-3

	n=105	n=71		n=71	n=53	

	1	2		2	3	
AvPAP, mmHg.	18.1±1.8	22.6±1.2#	<0.05	22.6±1.2#	27.4±1.2#	<0.05
FDDrv, mm	19,3±0.7	24,8±0.63#	<0.001	24.8±0.63#	31.6±0.9#	<0.001
FSDrv, mm	13.4±0.6	17,8±0.7#	<0.001	17.8±0.7#	25.9±0.8#	<0.001
ΔS% RV	30.5±2.3	28.2±2.4	–	28.2±2.4	18.0±2.4#	<0.01
FWTrv, mm	4.62±0.2*	5.29±0.1*	–	5.29±0.1*	5.80±0.2#	<0.05
H/D RV	0.19±0.01	0.19±0.01	–	0.19±0.01	0.17±0.02	–
FDD AD, mm	28.7±1.4	33.0±1.2#	<0.05	33.0±1.2#	37.2±1.6#	<0.05
RVET, ms	336±6.7	343±6.2	–	343±6.2	319±5.0	<0.05
PV Vmax m/s	0.72±0.01	0.81±0.01#	<0.001	0.81±0.01#	0.68±0.02	<0.01
TV DT, ms	204±3.2#	226±3.5#	<0.001	226±3.5#	200±3.2#	<0.001
TV IVRT, ms	81.2±2.4#	116.3±2.6#	<0.001	116.3±2.6#	95.8±3.8#	<0.001
TV Peak E, m/s	0.48±0.01	0.44±0.02*	–	0.44±0.02*	0.53±0.01	<0.01
TV Peak A, m/s	0.39±002*	0.42±0.02#	–	0.42±0.02#	0.37±0.02	–
TV E/A	1.24±002#	1.05±0.02#	<0.01	1.05±0.02#	1.43±0.01	<0.001

Remark: bold font and * and # designate values, trustworthily different from normal (p< 0.05 and p < 0.01. respectively).

The analysis of individual AvPAP values demonstrated that the most patients with verified CCP have values of this hemodynamic parameter in exceed of 20 mmHg. Simultaneously, it should be underlined that 19 of 71 patients of the 2^nd^ group (26.8%) and 5 patients of the 3^rd^ group (9.4%) had the PA pressure level less than 20 mmHg. On the other hand, 16 patients of the 1^st^ group without CCP signs (15.2%) the AvPAP value appeared to be higher than the stated threshold value. Thus, a direct dependence of presence or absence of cor pulmonale upon the AvPAP value measured while resting was not detected in many cases.

In assessment of these results one can assume that the PA average pressure value, measured in CCP patients only when they are resting, is far from precise demonstration of the increase extent of the pulmonary vessel resistance and the intensity of the relevant alterations in the heart muscle. It should be taken into account that the PA pressure level is to a certain extent dependent on the magnitude of the contractive capacity and the diastolic volume of the RV, presence or absence of tricuspid regurgitation, on the pre-tension of the RV and some other hemodynamic parameters (Brodskaya, Nevzorova, Geltser, & Katsyubriy, 2009; Han, Dong, Dimitropoulou, & Su, 2011; Pfeffer et al., 1992; Rydell-Tormanen, Risse, Kanabar, Bagchi, Czubryt, & Johnson, 2012), the latter being of special importance in the 3^rd^ group with de-compensated CCP.

The second most important sign typical for CCP patients is the growth of the *front wall thickness of the RV* (FWTrv). In our paper, as well as in works of other authors, as a criterion of a diagnostically significant FWT growth of the RV the values >5 mm were taken. The average FWTrv value in the patients of the 1^st^ group made up 4,62±0.2 mm, trustworthily different (p<0.05) from the relevant value in the reference group patients (3.86±0.3 mm), thus demonstrating a distinct tendency to myocardial mass growth of the PV long before the appearance of trustworthy *cor pulmonale* signs. Moreover, in 12 of 105 patients of the 1^st^ group (11.4%) FWTrv appeared greater than 5.0 mm. Nevertheless, it did not get a reason to assign the patients to the group with compensated CCP (2^nd^ group) in connection with the fact, that in accordance with the applied algorithm a diagnosis of RVH was considered probable only in case of compliance with all 3 clinical and instrumental criteria of CCP stated above.

Trustworthy differences of FWTrv from the norm were detected in the patients of the 2^nd^ (p<0.05) and especially of the 3^rd^ group (p<0.001), whose average values of this parameter made up accordingly 5.29±0.2 mm and 5.80±0.2 mm. Nevertheless, in 11 patients of the 2^nd^ group (10.5%) and 3 patients of the 3^rd^ group (5.6%) the values of FWTrv did not exceed 4.8 mm, and the diagnosis CCP was stated based on other clinical and echocardiographic signs of *cor pulmonale* (see below).

A number of principal parameters reflecting the heart re-modeling process in COPD patients, are *the final-systolic (FSDrv) and final-diastolic (FDDrv) dimension of the RV*. In COPD patients without CCP signs (1^st^ group) both systolic and diastolic dimensions do not trustworthily differ from those in the reference group patients (p<0.05), whereas in the patients of the 2^nd^ group with verified CCP moderate but statistically significant increase of FDDrv and FSDrv (p<0.001). In most cases of compensated CCP the values of these two parameters exceeded accordingly 22.0 and 15.0 ml, whereas in the patients of the 1^st^ group they appeared to be less than these values (p<0.001). The moderate increase of the dimensions of the RV in the patients of the 2^nd^ group is obviously stipulated by the effect of the compensatory mechanism of Frank-Starling, directed to the retaining of the stroke volume of the RV and the heart discharge. The proof for this is the absence in such patients of a trustworthy decrease of the parameter ΔS% RV (28.2±2.4%), which did not differ from that both in the patients of the reference group (31.7±2.5%), and in COPD patients of the 1^st^ group (30.5±2.3%). Simultaneously a rather high maximum blood flow velocity in the PA (PV Vmax) was retained, as well as the normal duration of the expulsion time of the RV (RVET).

The CCP de-compensation in the patients of the 3^rd^ group was accompanied by a still higher growth of FDDrv and FSDrv (p <0.001), which almost in all cases exceeded accordingly 26.0 and 20.0 mm. Thereby in patients with de-compensated CCP both diastolic and systolic RV dimension correlated in general well with the intensity of CHF. Simultaneously, in these patients a trustworthy decrease of ΔS% RV (up to 19, 4–15.0%) was registered, as well as the maximum blood flow velocity (PV Vmax) and the expulsion time (RVET), indirectly reflecting the progressive decrease of the contractive capacity of the RV and the onset of RV systolic function.

Thus, in the overwhelming majority of the cases the systolic and the diastolic RV dimensions grew in the COPD patients only when CCP was getting shape. It should be noted that the RV dimensions were even more trustworthy and reliable *cor pulmonale* marker than AvPAP and the front wall thickness of the RV. One should consider that technically the measurement of FDDrv and FSDrv is simpler than the calculation of the PA pressure and the measurement of FWTrv.

*The relative RV front wall thickness index* calculated as the proportion FWTrv vs. FDDrv (H/D) did not differ in the patients of the 2^nd^ group (0.19±0.01) from that of the reference group (0.20±0.01) and that of the 1^st^ group of COPD patients (0.19±0.01), being together with the trustworthy increase of the absolute FWTrv values an evidence of prevailing concentric hypertrophy signs in the 2^nd^ group.

In the patients of the 3^rd^ group with de-compensated CCP, in spite of the further myocardial mass growth of the RV, a statistically not trustworthy (p>0.05) tendency to the decreasing of this parameter (0.17±0.02) was observed, being an evidence of mostly eccentric RVH development in such patients.

It is well-known that for a normal pumping function of the ventricles the function of the atria is important. In this connection we studied the state of *atrium dextrum* (AD) in the COPD patients. As shown in Table 1, in the patients of the 1^st^ group the FDD AD value differed only slightly from the norm (p >0.05), although a not trustworthy tendency to a certain growth of this parameter as compared to the reference group (28.7±1.4 mm) was observed. More significant the AD dimension (33.0±1.2 mm) was in the patients with compensated CCP (p <0.001) what partially could be associated with the increase of the RV filling pressure, typical for patients with distinct compensatory hypertrophy of the RV and its diastolic dysfunction (see below). Thus, on the initial stage of *cor pulmonale* shaping, the increase of the AD dimensions was mostly of compensatory type reflecting the increase of the auricular pumping function.

In patients with de-compensated CCP (3^rd^ group) a greater dilatation of the AD (37.2±1.6 mm) was observed, the latter mostly stipulated by the growth of the central venous pressure (CVP) on the background of the progressive systolic and diastolic dysfunction of the RV. Of importance was also the frequent detection in these patients of the relative tricuspid valve insufficiency which was characteristic for patients with systolic ventricle dysfunction. In the patients of the 3^rd^ group structural changes of the right heart compartments (atrium and ventricle) were connected to one another, the evidence for it is a slight but trustworthy interaction between FDD AD and RV hollow dimension (r=0.42; p<0.01), as well as its wall thickness (r=0.38; p<0.05).

*The relative tricuspid valve insufficiency* was detected in the course of Doppler echocardiography in 21 CCP patients, thereby exclusively in patients with verified RVH and RV dilatation (FDDrv over 33 mm). The frequency of the tricuspid regurgitation was higher in de-compensated CCP patients (14 persons or 26.4%). In the patients of the 2^nd^ group the tricuspid valve insufficiency was detected only in 5 cases (7.0%). The difference is statistically trustworthy (p <0.05). For clear reasons, in the 3^rd^ group patients there was also a greater average extent if tricuspid regurgitation (2.46±0.7 vs. 1.32±0.5 st. in the patients of the 2^nd^ group), although statistically the difference was insignificant (p >0.05).

In the Doppler diagrams of the transtricuspid blood flow in the patients of the 1^st^ group a not trustworthy lowering of the amplitude of the E-peak and a statistically significant (p<0.05) increase of the A-peak height were observed. In this connection the E/A proportion in the patients of the 1^st^ group trustworthily decreased (p<0.001). Simultaneously an increase of the time (p<0.001) of the isovoluminal RV relaxation (IVRT) and the slow-down time of the early diastolic filling (DT) were registered.

In patients with compensated CCP (2^nd^ group) such changes were still more distinct: the IVRT duration reached 116.3±2.6 ms, DT – 226±3.5 ms, and the E/A proportion – 1.05±0.02 (p<0.001), that is, trustworthily different from the average values of the same parameters in the patients of the 1^st^ group (p<0.01).

In patients with distinct signs of heart failure and increased pressure gradient from atria to ventricles during the rapid filling phase the transformation of Type I of the diastolic dysfunction of the RV to Type II (restrictive), with distinguishing significant acceleration of the early diastolic filling of the ventricle (Peak E) and simultaneous decreased blood flow velocity during the systole of the atrium (Peak A). Thereby the proportion E/A increased, the phase of the isovoluminal relaxation was reduced (IVRT), as well as the slow-down time of the early diastolic filling (DT).

As a result, in general, in the 3^rd^ group moderate but trustworthy increase of the average values of Peak E (0.53±0.01 m/s vs. 0.44±0.02 m/s in the patients of the 2^nd^ group; p<0.01) and E/A (1.43±0.01 vs. 1.05±0.02 in the 2^nd^ group; p<0.001) and decrease of Peak A (0.37±0.02 m/s vs. 0.42±0.02 m/s; p>0.05), of IVRT (95.8±3.8 ms vs. 116.3±2.6 ms; p<0.001), and DT (200±3.2 ms vs. 226±3.5 ms; p<0.001) were observed, reflecting a more distinct tendency to the generation of the restrictive type of the diastolic dysfunction of the RV, commonly known as the evidence of an essential increase of the ventricle filling pressure and considered to be rather unfavorable in terms of prognosis.

## 5. Conclusions

1) In the earliest stages of morphological and functional heart remodeling, long before the manifestation of commonly known signs of CCP (hypertrophy and/or dilatation of the RV), in COPD patients moderate abnormality of the diastolic function of the RV of Type I (slowed-down relaxation) and the increase of the RV front wall thickness are observed, not reaching the diagnostically significant level (5.0 mm). In this stage of CCP generation in COPD patients only a not trustworthy tendency to increased AvPAP while resting, and AD (p >0.1) is observed.
2) The shaping out of compensated CCP (in the patients of the 2nd group) is generally characterized by a trustworthy growth of AvPAP (>20 mmHg), the growth of the front wall of the RV (>5 mm), as well as the growth of the diastolic and systolic dimensions of the RV hollow (>22 mm and 15 mm respectively) whereas normal values of the relative front wall thickness index (H/D) are retained.
3) The de-compensation of CCP and the development of RV CHF is accompanied by a still greater increase of AvPAP (≥25 mmHg while resting), of FDDrv and FSDrv (>26 mm and 20 mm respectively), of the RV front wall thickness (>5 mm), of AD (>35 mm), as well as by the reduction of the extent of the vestibular-distal RV shortening (ΔS %) (<23%), of the maximum blood flow velocity (PV Vmax), and the duration of the expulsion period of blood from the RV (RVET), being an indirect evidence of the progressive reduction of the myocardial contractive capacity of the RV and the onset of its systolic dysfunction.
